# Glioblastoma recurrence followed by newly diagnosed small cell lung cancer: a case report on personalized concurrent chemoradiotherapy and therapeutic considerations

**DOI:** 10.3389/fonc.2026.1793568

**Published:** 2026-04-22

**Authors:** Xue Ren, Feng Shang, Defu Yang, Ying Yan, Ying Xu

**Affiliations:** Department of Radiation Oncology, General Hospital of Northern Theater Command, Shenyang, Liaoning, China

**Keywords:** case report, concurrent chemoradiotherapy, glioblastoma, personalized treatment, second primary tumor, small cell lung cancer

## Abstract

Glioblastoma (GBM) and small cell lung cancer (SCLC) are both highly aggressive malignancies, and their co-occurrence is extremely rare, posing significant therapeutic challenges. We report a 50-year-old male diagnosed with right temporal GBM (WHO grade IV) in 2014, who underwent surgery and the standard Stupp regimen (radiotherapy with temozolomide, TMZ), achieving a progression-free survival (PFS1) of 42 months. During re-irradiation with TMZ for the first GBM recurrence, a second primary tumor—SCLC (cT3N2M0, Stage IIIB)—was identified 45 months after the initial diagnosis. For this rare dual malignancy, an individualized concurrent chemoradiotherapy approach was implemented: continued cranial radiotherapy with TMZ alongside etoposide plus platinum (EP) chemotherapy and subsequent palliative thoracic radiotherapy for the lung tumor. Treatment was well-tolerated with no grade ≥3 adverse events, resulting in partial response of the pulmonary lesion and stable intracranial disease for 10 months. The patient experienced a PFS2 of 10 months after the second GBM recurrence and a PFS of 13 months for SCLC, with an overall survival (OS) of 58 months from initial GBM diagnosis—substantially exceeding the typical prognosis for either tumor alone. This case suggests that an individualized concurrent chemoradiotherapy strategy may achieve dual tumor control and confer meaningful survival benefits in patients with recurrent GBM and metachronous SCLC.

## Introduction

GBM is the most common and aggressive primary brain tumor in adults. Despite treatment involving maximal safe resection followed by concurrent radiotherapy and TMZ chemotherapy, the median OS remains limited to approximately 15–20 months (1). SCLC is a highly aggressive neuroendocrine malignancy, accounting for about 15% of all lung cancers, with a median OS of only around 12 months in extensive-stage disease (2). The co-occurrence of these two highly malignant tumors as independent primary cancers in the same patient is exceedingly rare. Local recurrence of GBM often necessitates repeat radiotherapy and chemotherapy (3), while SCLC management relies on platinum-based systemic chemotherapy and radiotherapy (4). When both conditions coexist, clinicians face multiple challenges, including determining the optimal treatment sequence, managing potential interactions and overlapping toxicities between different chemotherapy regimens (e.g., TMZ and etoposide/platinum), and addressing long-term risks associated with repeated or extensive radiation fields. Currently, no standard therapeutic guidelines exist for this complex clinical scenario (5). In the era of precision oncology and multidisciplinary collaboration, personalized management of rare and complex cases has gained increasing attention. Detailed reporting of such cases, along with systematic analysis of treatment decisions, survival outcomes, and adverse events, is crucial for accumulating clinical experience, exploring cross-cancer synergistic treatment strategies, and guiding future clinical research directions (6).

## Case description

**Figure 1:**
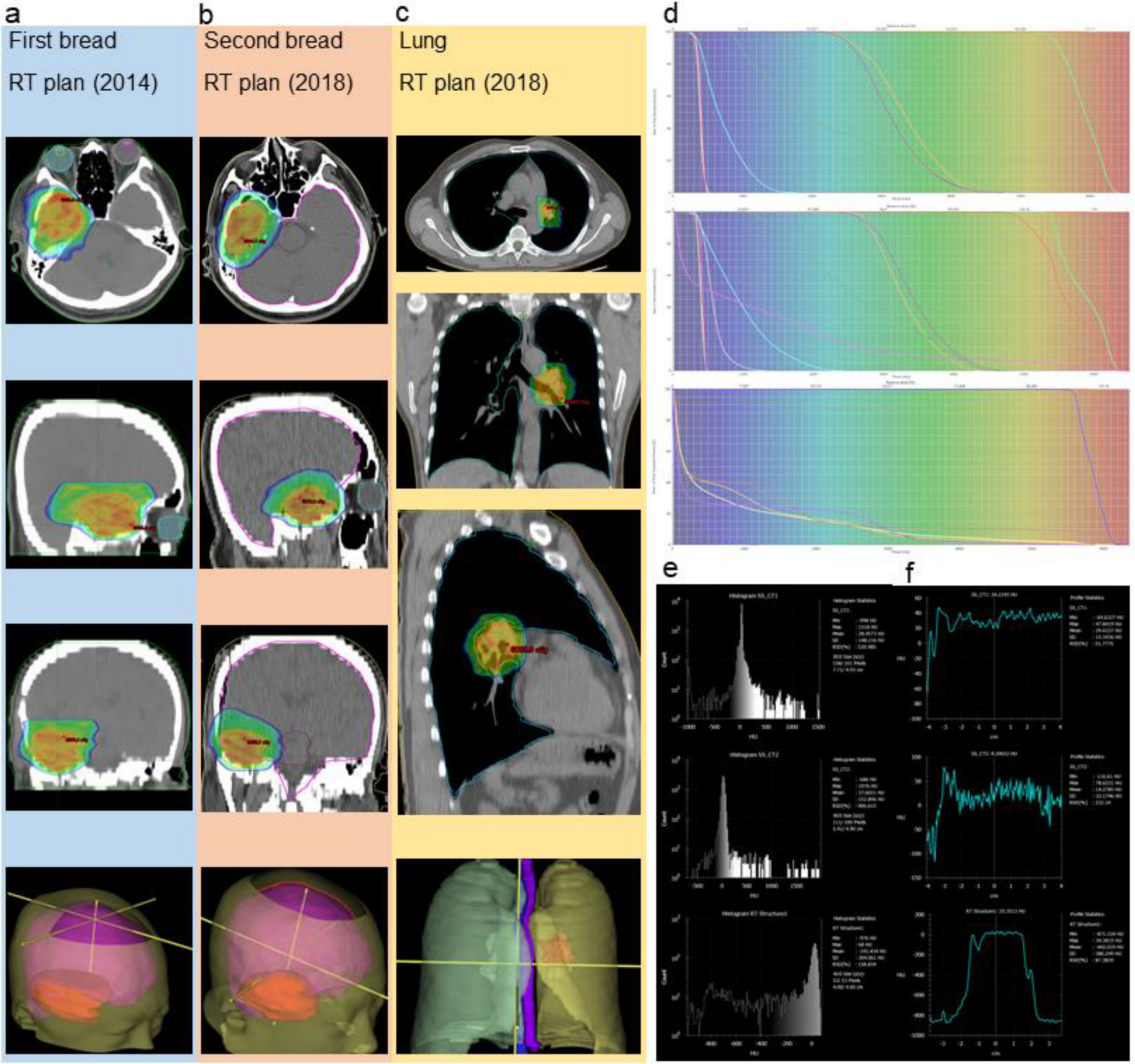
Radiotherapy treatment plans. **(a)** First bread RT plan (2014). **(b)** Second bread RT plan (2018). **(c)** Lung RT plan (2018). **(d)** Dose-volume histograms (DVH) for all three plans. **(e)** Histograms of Hounsfield unit distribution. **(f)** Profiles along tumor center.

**Figure 2:**
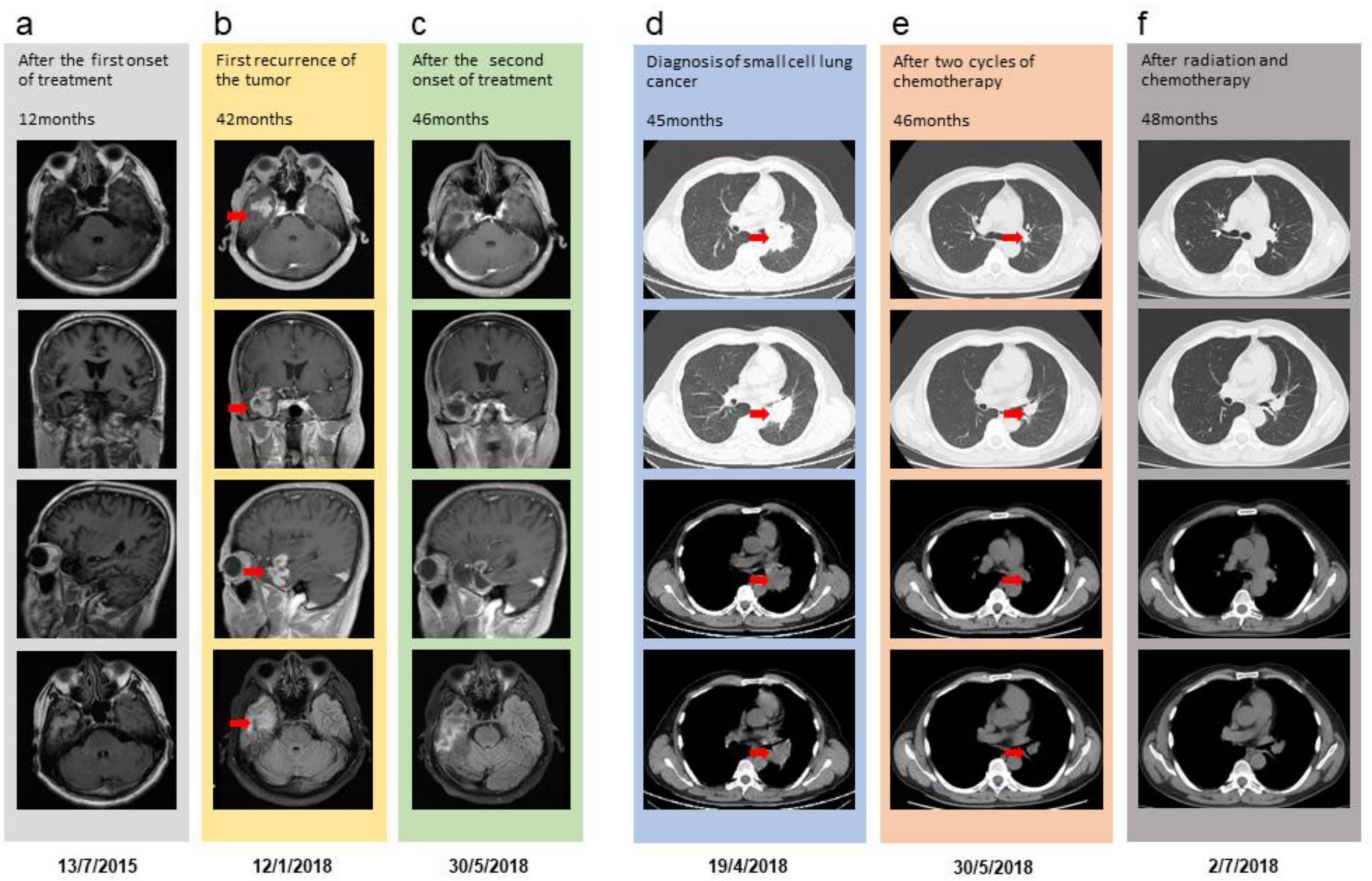
Clinical course of the patient. **(a)** Twelve months after initial diagnosis, MRI (T1-enhanced, axial/coronal/sagittal; T2-FLAIR axial) showing no evidence of disease. **(b)** Forty-two months after initial diagnosis, MRI showing recurrent right temporal lobe tumor (red arrow). **(c)** Forty-six months after initial diagnosis, MRI post-chemoradiotherapy showing stable intracranial disease. **(d)** Forty-five months after initial diagnosis, chest CT showing left hilar mass (red arrow) and mediastinal lymphadenopathy. **(e)** Post-chemotherapy (2 cycles) CT showing marked shrinkage of left hilar mass (red arrow). **(f)** Post-concurrent chemoradiotherapy CT showing stable pulmonary findings. (MRI parameters: 3.0T, slice thickness 5mm; CT parameters: slice thickness 3mm).

A 50-year-old man with no remarkable past medical history presented with a Karnofsky Performance Status (KPS) score of 90, a body surface area of 1.71 m^2^, and no family history of cancer. On July 15, 2014, he underwent gross total resection of a right temporal space-occupying lesion in the Department of Neurosurgery at our institution. Histopathological examination confirmed giant cell GBM of the right temporal lobe (WHO grade IV). Molecular testing was not performed at the time of initial diagnosis. In accordance with the 2014 National Comprehensive Cancer Network (NCCN) guidelines, the patient received standard postoperative cranial radiotherapy with concurrent and adjuvant TMZ. The initial radiotherapy plan is shown (**Figure 1a**). Concurrent chemotherapy was administered according to the Stupp regimen with TMZ at 75 mg/m^2^, followed by six cycles of adjuvant TMZ at 200 mg/m^2^. Treatment was well tolerated, and no severe adverse events affecting daily functioning or occupational activities were observed. Follow-up magnetic resonance imaging (MRI) obtained 1 year after surgery is shown (**Figure 2a**). At 42 months after the initial diagnosis, the patient developed headache. Follow-up MRI demonstrated a recurrent right temporal lesion measuring approximately 3.7 × 2.8 × 2.5 cm, with marked heterogeneous enhancement (**Figure 2b**). In line with the 2018 NCCN recommendations, he underwent repeat surgical resection, achieving gross total removal with negative margins. Histopathological analysis again confirmed giant cell glioblastoma, WHO Grade IV. Molecular profiling at recurrence revealed MGMT promoter methylation, intact 1p/19q, and wild-type IDH1/2. According to the NCCN Clinical Practice Guidelines in Oncology for Central Nervous System Cancers (Version 1.2017) (7), re-irradiation may be considered for selected patients with recurrent GBM, particularly in combination with systemic therapy. Based on this recommendation, the patient underwent cranial re-irradiation with concurrent TMZ using the Stupp regimen (**Figure 1b**). During treatment for recurrent intracranial disease, at 45 months after the initial diagnosis, the patient was diagnosed with a second primary malignancy, namely SCLC. He presented with chest tightness and dyspnea. Chest computed tomography (CT) revealed a left hilar and mediastinal mass measuring approximately 6.7 × 4.7 cm, causing compression and narrowing of the adjacent bronchus, together with enlarged mediastinal lymph nodes measuring approximately 5.4 × 4.0 cm (**Figure 2d**). Bronchoscopic biopsy confirmed small cell undifferentiated carcinoma with neuroendocrine differentiation. The disease was staged as cT3N2M0, stage IIIB. The SCLC was diagnosed in October 2017, corresponding to 45 months after the initial GBM diagnosis. The treatment sequence was as follows: two cycles of EP chemotherapy were administered between November and December 2017; palliative thoracic radiotherapy was then delivered in 26 fractions between January and February 2018. During the same period, cranial re-irradiation and oral TMZ continued from December 2017 to February 2018, resulting in an overlap of approximately 4 weeks between the two chemoradiotherapy courses. The thoracic radiotherapy plan is presented (**Figure 1c**). After two cycles of chemotherapy, repeat chest CT demonstrated marked regression of the left hilar mass to approximately 1.6 × 1.5 cm, while the enlarged mediastinal lymph nodes decreased to approximately 1.8 × 2.8 cm (**Figure 2e**). Meanwhile, the intracranial lesion remained stable (**Figure 2c**). Subsequent imaging after completion of concurrent chemoradiotherapy showed sustained stability of both the pulmonary and mediastinal lesions. The patient then continued oral TMZ and EP chemotherapy, ultimately completing six cycles of adjuvant TMZ and three cycles of EP chemotherapy. Overall, treatment was well tolerated. Adherence exceeded 95%, and all planned courses of radiotherapy and chemotherapy were completed. Adverse events included grade 1 leukopenia, grade 1 fatigue, grade 1 radiation dermatitis, grade 1 radiation pneumonitis, and grade 1 esophagitis. All toxicities were self-limited or adequately controlled with symptomatic treatment. No grade 3 or higher adverse events occurred. During this period, the patient maintained a KPS score of 80. In November 2018, 10 months after the second surgery, the patient developed clumsiness of the left upper limb, hearing decline, visual deterioration, and gait instability. Multidisciplinary team (MDT) evaluation concluded that these findings represented a second intracranial radiographic progression event. Combined TMZ and bevacizumab therapy was recommended. However, after one cycle of treatment, symptom relief was minimal. Owing to financial constraints, the patient and his family declined further antitumor therapy and opted for palliative care at a local hospital. The patient died on May 30, 2019, and death was considered attributable to progression of the brain tumor. Chest CT performed before death showed that the pulmonary lesions remained stable. The first PFS of GBM was 42 months, and the PFS after the second intracranial progression event was 10 months, yielding an OS of 58 months. For SCLC, both PFS and OS were 13 months. Details of the three radiotherapy plans are summarized in **Table 1**, and dosimetric evaluations are shown (**Figure 1**).

**Table 1:** Summary of radiotherapy courses and treatment-related toxicity.

Radiotherapy site	Indication	Fractionation schedule	Electron energy	Total dose	Guiding guidelines	Treatment outcome	Adverse toxicity (CTCAE Grade)
Bread (2014)	Postoperative right temporal GBM	2Gy*30f	6MeV	60Gy	NCCN 2014	Completed as planned	Grade 1 radiation dermatitis
Bread (2018)	Postoperative recurrent GBM	2Gy*30f	6MeV	60Gy	NCCN 2018	Completed as planned	Grade 1 fatigue
Lung (2018)	Left lung SCLC	2Gy*26f	6MeV	52Gy	NCCN 2018	Completed as planned	Grade 1 radiation pneumonitis and esophagitis

## Discussion

GBM and SCLC are both highly aggressive malignancies. Each is associated with substantial therapeutic challenges. In the present case, however, the patient faced an unusual and clinically complex circumstance: the simultaneous management of both tumors. He initially underwent gross total resection for a right temporal GBM, followed by adjuvant radiotherapy and TMZ-based chemotherapy in accordance with NCCN recommendations. After 40 months of disease stability, the GBM recurred, and the patient subsequently received repeat surgery, re-irradiation, and continued TMZ maintenance therapy. During this treatment course, he was diagnosed with a second primary malignancy, namely SCLC. While continuing oral TMZ, he also underwent etoposide–platinum (EP) chemotherapy and palliative thoracic radiotherapy. The coexistence of these two aggressive malignancies created a distinct therapeutic dilemma. The standard management of high-grade glioma relies on maximal safe resection, radiotherapy, and TMZ, whereas SCLC generally requires intensive systemic chemotherapy combined with thoracic irradiation. According to NCCN guidelines, EP-based regimens may be considered a therapeutic option in recurrent GBM when preferred regimens are ineffective or not tolerated (8). In the present case, the EP regimen not only achieved disease control for SCLC but also served as a second-line systemic treatment for recurrent GBM. Conversely, TMZ has also been explored in the treatment of brain metastases from SCLC, with evidence suggesting that TMZ combined with whole-brain radiotherapy may improve objective response rates and prolong survival (9). Therefore, the combined use of these two chemotherapy regimens may have provided dual antitumor activity in a patient harboring both high-grade glioma and SCLC.

A biologically plausible synergistic mechanism may also exist between TMZ and the EP regimen. TMZ induces DNA methylation damage, particularly O6-methylguanine lesions, whereas platinum compounds generate DNA adducts; both agents disrupt DNA replication and transcription and may act synergistically through distinct DNA damage response pathways. Etoposide, through inhibition of topoisomerase II, may further impair DNA repair and enhance chemosensitivity (10). On the other hand, cranial radiotherapy can disrupt blood–brain barrier integrity and increase central nervous system exposure to systemically administered agents, thereby potentially exacerbating neurotoxicity (11). In this case, however, with strict dose control and regular neurologic assessment, no grade ≥3 neurologic toxicity was observed, suggesting that this combined strategy may be feasible and acceptably safe under close surveillance. Following the second intracranial radiographic progression event, bevacizumab-based treatment yielded limited benefit. Several explanations may account for this observation. Acquired therapeutic resistance in GBM involves complex molecular mechanisms, including specific microRNA signatures (e.g., miR-21 and miR-10b) associated with bevacizumab resistance (12). The intrinsically modest activity of bevacizumab monotherapy in recurrent GBM achieves objective response rates of only 20%–30%, and recent studies confirm median survival of approximately 6–10 months after first progression (13). Furthermore, the prior multimodal treatment pressure in this case may have accelerated the expansion of resistant subclones. Potential alternative strategies might have included immune checkpoint inhibitors (although anti-PD-1 monotherapy has shown limited survival benefit in recurrent GBM) (13), targeted therapy combinations, or enrollment in clinical trials investigating novel agents such as MDM2 inhibitors (13) or approaches to overcome anti-angiogenic therapy resistance (12). received Importantly, the overall treatment strategy in this case remained broadly consistent with the core principles of the 2025 NCCN recommendations, namely maintenance of the standard radiotherapy-plus-TMZ backbone for GBM and use of EP chemotherapy combined with thoracic radiotherapy for SCLC, while allowing individualized adaptation within a guideline-based framework. In November 2018, the patient experienced a second intracranial radiographic progression event. MDT assessment, based on the Response Assessment in Neuro-Oncology (RANO) criteria, supported progression on the basis of worsening clinical symptoms together with enlargement of T1-enhancing and T2/FLAIR abnormalities. The neuroradiology team recommended postoperative baseline MRI as the reference standard and suggested that magnetic resonance spectroscopy (MRS), diffusion-weighted imaging (DWI), and PET-CT might have been helpful in distinguishing true progression from pseudoprogression. Neurosurgeons concurred that the imaging findings fulfilled RANO criteria for progression. However, because the patient and his family declined repeat surgery or biopsy, pathological confirmation—the diagnostic gold standard—could not be obtained. Furthermore, because subsequent imaging follow-up was refused, dynamic evolution of the lesion could not be assessed. Accordingly, this event may have represented true progression, pseudoprogression, or treatment-related brain injury, and a definitive distinction could not be established.

This case underscores the complexity of managing rare dual primary malignancies. Through close multidisciplinary collaboration, we implemented an individualized strategy integrating radiotherapy, chemotherapy, and targeted therapy, with particular attention to target delineation, dose distribution, and organ-at-risk sparing. Encouraging short-term outcomes were achieved: the pulmonary lesions regressed, intracranial disease remained stable for a period, and treatment-related toxicities were acceptable. Nevertheless, long-term follow-up was limited because treatment was discontinued for financial reasons, and the patient died 6 months after therapy cessation, with death considered most likely attributable to progression of the brain tumor. To our knowledge, this is the first reported clinical case describing the concurrent use of TMZ plus cranial re-irradiation and EP chemotherapy plus palliative thoracic radiotherapy in a patient with recurrent GBM and synchronous second primary SCLC. Compared with previously reported cases of coexisting GBM and SCLC, in which median OS has ranged from 8 to 24 months (14), the present patient achieved a substantially longer OS of 58 months. This favorable outcome suggests that a carefully selected, individualized strategy of concurrent cranial and thoracic chemoradiotherapy may provide meaningful survival benefit in such rare and highly challenging clinical scenarios. Notably, dual tumor control was achieved without any grade ≥3 adverse events, further supporting the feasibility and tolerability of this approach.

## Clinical takeaways

Several clinical lessons may be drawn from this case. First, multidisciplinary collaboration is essential; continuous MDT involvement from diagnosis through follow-up provides the foundation for comprehensive management of complex dual malignancies. Second, individualized concurrent chemoradiotherapy appears feasible; under intensive monitoring, cranial re-irradiation plus TMZ and thoracic radiotherapy plus EP chemotherapy may be delivered concurrently with acceptable toxicity while achieving disease control at both sites. Third, the combination of TMZ and EP may confer synergistic benefit, and a dual-chemotherapy strategy directed against both tumors may contribute to prolonged survival. Fourth, close toxicity surveillance is critical; routine assessment using Common Terminology Criteria for Adverse Events (CTCAE) version 5.0 criteria enables early recognition and management of low-grade adverse events and may help prevent severe complications.

## Conclusion

In summary, this report provides valuable experience in the individualized management of GBM coexisting with SCLC. It highlights the critical role of MDT and the need for innovative, flexible therapeutic approaches when confronting such complex clinical scenarios. Further research and systematic reporting of similar cases are warranted to refine treatment strategies and improve outcomes for these rare and challenging patients.

## Data Availability

The original contributions presented in the study are included in the article/supplementary material. Further inquiries can be directed to the corresponding authors.
